# Commentary: Local sympathetic nerve depletion does not alter vitiligo progression in a mouse model

**DOI:** 10.3389/fmed.2025.1590092

**Published:** 2025-07-28

**Authors:** Yubin Peng, Tao Wang

**Affiliations:** ^1^Department of Dermatology, Peking Union Medical College Hospital, Chinese Academy of Medical Sciences and Peking Union Medical College, Beijing, China; ^2^State Key Laboratory of Complex Severe and Rare Diseases, Beijing, China; ^3^National Clinical Research Center for Dermatologic and Immunologic Diseases, Beijing, China

**Keywords:** skin disease, vitiligo, sympathetic nerve, commentary, mouse model

## 1 Introduction

The recent article by Hu et al. has challenged the existing hypothesis regarding the potential pathogenic role of sympathetic nerves in vitiligo. Their study randomized mice into two groups: a chemical sympathectomy group, which underwent sympathetic denervation via 6-hydroxydopamine (6-OHDA), and an untreated control group. Both groups were subsequently induced with vitiligo using the melanoma–regulatory T cell (Treg)-induced vitiligo model. Epidermal melanocytes and CD8^+^ T cells of tail skin were quantified before and after induction. The authors discovered that tyrosine hydroxylase (TH)^+^ sympathetic nerve fibers in both back and tail skin were significantly depleted by 6-OHDA treatment. In contrast, the number of tail epidermal melanocytes and CD8^+^ T cells was comparable between the two groups, before and after vitiligo induction. Contrary to the prevailing hypothesis (1), they reported that sympathetic nerve activity did not affect vitiligo progression.

## 2 Discussion

Although groundbreaking in its contribution, we identified some limitations in the study. First, although their melanoma-Treg-induced vitiligo mice indeed showed reduced numbers of melanocytes and CD8^+^ T cells, photographic evidence of white or gray skin and hair is necessary to satisfy the critical diagnostic criteria for observable hypopigmented lesions and to validate the authenticity of this model.

Importantly, this model depletes both Tregs and other CD4^+^ T cells such as helper T (Th)1 cells, making it unsuitable for a CD4^+^-related study (2). Consequently, this model omits immune responses mediated by Th1 and Th17, which directly contribute to melanocyte destruction through interferon (IFN)-γ and interleukin (IL)-17 (3). While this model emphasizes CD8^+^ T cells, reflecting their direct killing effect and the efficacy of current targeted treatment, it overlooks the indispensable role of CD4^+^ T cells in immune regulation. This restriction may have led to a skewed interpretation of the results and a potentially biased conclusion.

A previous study demonstrated that stress-triggered sympathetic nerve activation leads to hair graying by depleting melanocyte stem cells (MeSCs), and identified sympathetic nerve activation as the critical step in this process, independent of immune-mediated mechanisms (4). To address the potential oversight of stress-related sympathetic nervous system involvement, we recommend incorporating stress induction into future experimental designs. Including a stress stimulus could help clarify whether sympathetic nerve activity plays a functional role in disease onset or severity in this model.

Furthermore, it has also been hypothesized that sympathetic nerves modulate MeSC activity, melanocyte migration, or pigment production under conditions independent of the hair cycle (4). It has been suggested that, while MeSCs may be depleted, differentiated melanocytes and melanin synthesis are not directly affected, implying that differentiated melanocytes might not decline immediately following MeSC loss. When determining the optimal time point to quantify melanocytes, this delayed effect should be considered. Repeated measurements at multi-day intervals could help capture dynamic changes more accurately and improve the reliability of the analysis.

To investigate the potential role of the sympathetic nervous system in vitiligo development through neurotransmitter signaling mechanisms, particularly involving norepinephrine, a final analysis should extend beyond existing cell quantification to include measurements of circulating and local neurotransmitter levels.

Regarding the experimental design, adding a sympathetic nerve activation group using beta-adrenergic agonists, in contrast to the existing depletion group, may facilitate a more accurate assessment of the role of the sympathetic nervous system in vitiligo. Finally, to exclude false-negative results and confirm the validity of the experimental system, we recommend that the authors consider including another positive control group. For example, treatment with IFN-γ or tumor necrosis factor-alpha (TNF-α), both of which are known to exacerbate vitiligo (5), could serve as positive controls. Additionally, should circumstances permit, augmenting the sample size of mice would enhance statistical power and improve the robustness of the findings.

In summary, we hope that our feedback on this creative, pioneering study of vitiligo pathogenesis, particularly our suggestions regarding experimental timing and optimization of the modeling framework, will help inform and inspire future research endeavors in this field.
